# Existence, triggers, and coping with chronic sorrow: a qualitative study of caretakers of children with sickle cell disease in a National Referral Hospital in Kampala, Uganda

**DOI:** 10.1186/s40359-018-0263-y

**Published:** 2018-10-20

**Authors:** Connie Olwit, Maureen Mugaba, Charles Peter Osingada, Rose Chalo Nabirye

**Affiliations:** Department of Nursing, College of health sciences, Makerere University, P.O.BOX. 7072, Kampala, Uganda

**Keywords:** Chronic sorrow, Experiences, Caretakers, Caregivers, Sickle cell disease, Low income country, Uganda

## Abstract

**Background:**

Worldwide, sickle cell disease is recognized as one of the major causes of morbidity and mortality. Caregivers and patients with such chronic illnesses experience economic, physical, social and psychological distresses which may lead to chronic sorrow. Chronic sorrow is viewed as a normal reaction to loss, however it can progress to a pathological state such as depression if the coping styles are ineffective. Therefore, the aim of this study was to explore the existence of chronic sorrow, triggers and coping with grief related feelings among caretakers of children with sickle cell disease.

**Methods:**

A descriptive qualitative study was conducted. Twelve in-depth interviews were conducted with eligible participants who were purposively selected. Deductive thematic analysis methods were used for data analysis.

**Results:**

Many (9 out of 12) of the caretakers experienced chronic sorrow. The grief related feelings were triggered by health worker related, disease related and support related factors. Caretakers used both external and internal coping strategies. External support was derived from community, family and health facility. Internal coping strategies were behavioral and cognitive.

**Conclusion:**

Caretakers of children with sickle cell disease experienced chronic sorrow and employed both internal and external coping strategies to deal with it, which could be either effective or ineffective. This study recommends that health workers should routinely screen for chronic sorrow among caretakers of children with sickle cell disease and assist caretakers to strengthen effective coping strategies to ameliorate the negative effects of chronic sorrow.

## Background

It is estimated that over 300,000 babies worldwide are born with Sickle Cell Disease (SCD) annually [1]. In Uganda, 70–80% of children with SCD die before the age of 2 years and those who survive live a compromised quality of life due to the effects of the disease [2]. Children with SCD usually experience vaso -occlusion which results in pain, anemia, stroke, leg ulceration, organ damage and early mortality [3]. These patients experience recurrent painful crises, acute chest syndrome, priapism and other complications such as neurocognitive impairment and acute silent cerebral infarcts among others [3, 4]. These complications cause numerous hospitalizations and an alteration in body image in these patients [5]. Caretakers of children with SCD have reported challenges associated with the provision of physical, psychological and social care [6, 7]. Repeated hospitalization, altered body image and changes that come with the disease may affect self-esteem and the social life of both caretakers and individuals with SCD [6]. This may result in psychological distress resulting in a phenomenon called Chronic Sorrow.

Chronic sorrow is defined as the periodic recurrence of permanent, pervasive sadness or grief related feelings associated with significant loss [8]. In the case of SCD, chronic sorrow may result from a disparity between a parent’s/caretaker’s expectations of a healthy child and the reality of having a child with SCD. Disparity in this case is defined as the difference between the children who have SCD and those without. Although it is viewed as a normal reaction, chronic sorrow can progress to a pathological state such as depression if coping styles are ineffective [9]. Recognizing chronic sorrow among caretakers of patients with SCD and maladaptive coping strategies is useful in ensuring that effective strategies are designed to deal with negative effects in a timely manner [8, 9].

In recent studies, chronic sorrow has been described among caregivers of children with various chronic conditions such as mental illness, diabetes, epilepsy, alagille syndrome and cerebral palsy [10–15]. However, most of these studies were conducted in high income countries. Few studies have been conducted in low and middle-income countries to explore chronic sorrow. Therefore, this study describes the existence of chronic sorrow, triggers of grief related emotions and coping strategies used by caretakers of children with sickle cell disease in a low-income country.

## Methods

### Study setting

The study was conducted at the Sickle Cell Clinic of Mulago National Referral Hospital (MNRH). Mulago is one of two national referral hospitals in Uganda and also serves as the teaching hospital for Makerere University College of Health Sciences and several other training institutions in Uganda. The hospital has a bed capacity of 1500 and an annual inpatient turnover of 120,000 [2]. The sickle cell clinic runs cost-free daily services and receives about 250–300 patients each week. Mulago was chosen as the study site because it is the only public institution with a specialized clinic for SCD patients.

### Study design

This study employed a descriptive qualitative design using face to face in-depth interview method for data collection. Twelve in-depth interviews (IDI) were conducted. This sample size was guided by the principle of saturation where data was collected until the researchers could not find new information from the participants. Participants included adult caretakers of children diagnosed with SCD aged one to 18 years, who had taken care of the same patient for at least 1 year. The study excluded caretakers whose children were in sickle cell crisis at the time of data collection.

### Sampling and data collection

Purposive sampling was used to select study participants who were articulate, could easily and clearly describe their experiences and consented to participate in the study. In order to have a rich description of the phenomenon, both men and women were purposively selected to participate in the study.

Caretakers were approached and asked whether they were interested in participating in a study about SCD. An explanation of the research was provided and written informed consent was obtained from those caretakers who agreed to be interviewed. The in-depth interviews were conducted by nurses who were trained for 2 days prior to data collection. In order to limit challenges associated with power differences, the interviewers were neither dressed in uniform nor were they staff of the sickle cell clinic. Privacy was ensured during the interviews by interviewing participants in a private room. The interviews were conducted in Luganda which is the most commonly spoken language in the central part of Uganda. All interviews were audio recorded with the participants permission.

The interviews were guided by the Burke/ Nursing Consortium for Research on Chronic Sorrow (NCRCS) Interview guide for caregivers which contains 16 open ended questions [16]. It is designed to evaluate the occurrence of chronic sorrow (1–6 questions), intensity of the sorrow, milestones at which chronic sorrow occurred, individualized coping factors, support from others and advice caregivers received from others. The interview guide was translated into Luganda and pretested to ensure that the meanings of the questions were clear and easily understood in Luganda. This helped to assess the flow of the interview, the probing questions and how long the interviews would take. Pre-testing was done with 2 caretakers of cancer patients in Mulago Hospital. The interview guide was modified appropriately prior to data collection. The interviewers established rapport with the respondent to build trust and encourage free expression of emotions. Data were collected from February to March 2016. This study adhered to the Helsinki Declaration of 2013 and ethical clearance to conduct the study was obtained from both Makerere University School of Health Sciences and Mulago Hospital Institutional Review Boards.

### Analysis

Audio taped in-depth interviews were transcribed verbatim and translated into English. The interviews were read several times to obtain a sense of the entire script. Data was analysed using deductive thematic analysis. Deductive thematic analysis was used because the researchers conceptualised the study from a theory of chronic sorrow where they narrowed down to themes were identified [8]. The middle range theory of chronic sorrow gives elaborate explanation about this phenomenon. The theorists explain that when there is a single or on-going significant loss, it results in disparity where there is a difference between a child with SCD and a healthy child. In this study the significant loss was having a child with SCD. The disparity leads to chronic sorrow which is pervasive, periodic or permanent in nature. When experiencing chronic sorrow, individuals express sadness or grief related feelings which are triggered by different factors such as the when the child is sick or stigma related to the loss. When chronic sorrow is experienced, caretakers employ internal or external coping strategies which may or may not be effective. The effective methods shorten the period of sorrow and increase comfort of the caretaker. The ineffective methods increase discomfort among caretakes with sorrow/ grief related feelings. The whole process is repeated when a trigger event occurs which make chronic sorrow cyclic.

During analysis, researchers identified four main themes from the framework namely; disparity, chronic sorrow, triggers and coping strategies. The texts of the transcripts were read and divided into condensed meaningful units, abstracted and labelled with a code. The codes were compared based on similarities and differences and were then sorted into categories. Finally, the categories were placed under different themes identified from the framework of the theory of chronic sorrow. The analysis was done by four individuals who developed codes and categories independently, this was proceeded by a group discussion to compare codes from each script and get consensus.

### Trustworthiness

To increase the rigour of the study, time was taken to build rapport and trust with informants; this helped the informants to feel at ease and share their experiences freely and in depth. There was also continuous non-threatening observation of nonverbal clues.

To facilitate transferability, a clear and distinct description of the characteristics of study participants, data collection methods and the process of analysis was done. A rich and robust presentation of the findings with appropriate verbatim participant quotations was done.

## Results

### Socio demographic characteristics

Most participants were female (83%) and married (67%). Most caretakers were parents of the children with SCD and most of the children had lived with SCD for 3 to 5 years (67%) (Table 1).

**Table 1:** Socio demographic characteristics of the participants

Variable	Frequency
Age (years)	
< 30	2
30–39	8
> 40	2
Sex	
Female	10
Male	2
Religion	
Catholic	3
Protestant	5
Moslem	3
Born again	1
Marital status	
Married	8
Separated/divorced	3
Single	1
Level of education	
Primary	3
Secondary	5
Tertiary	2
Relationship with child	
Biological Child	11
Not Biological Child	1
Duration with SCD (years)	
< 3	3
4–5	8
> 6	1

**Fig. 1:**
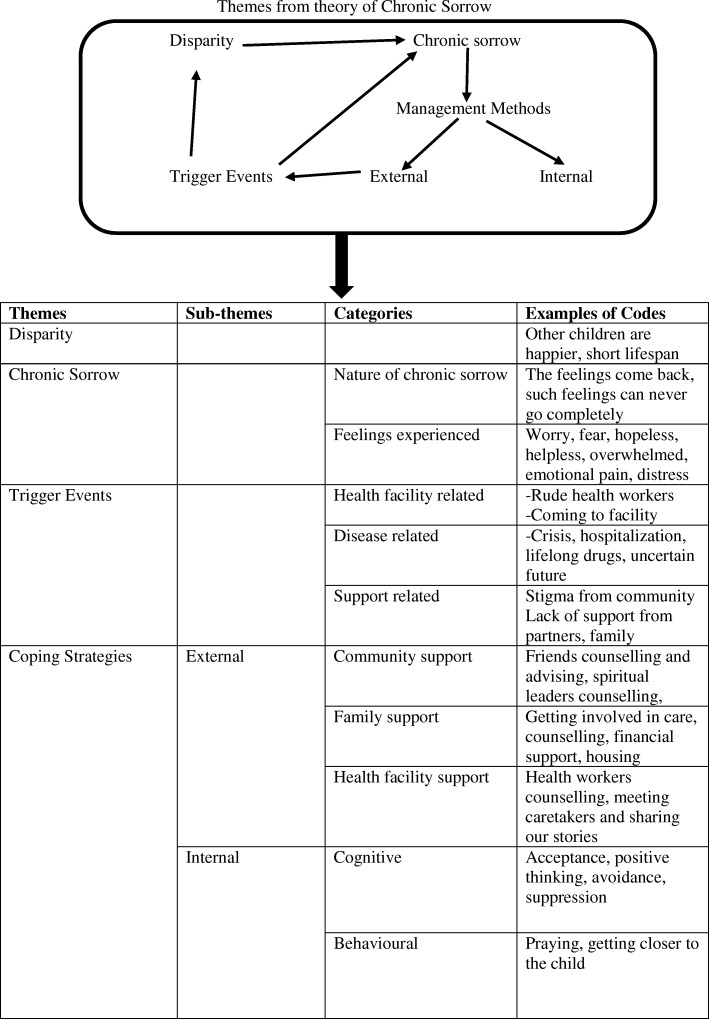
Showing analysis flow

Four main themes were identified from the middle range theory of Chronic Sorrow; disparity, chronic sorrow, trigger factors, and coping strategies. The findings are presented under these four main themes (See analysis flow in Fig. 1).

### Disparity

Disparity is the difference created by those experiencing the loss and those without loss. In this case, having a child with SCD was on-going loss. Caretakers experienced disparity when they compared their children’s growth to those who did not have SCD (4 out of 12). Due of the nature of SCD, the children with SCD fail to thrive as the normal children, this precipitated sadness among caregivers. This was noted by a caretaker who said;

*“Life is not good…because she has never regained her health as she used to be… even after giving medicine every day, she is not like other family members.” IDI 10*

Other caregivers experienced disparity when they compared their child’s happiness to that of other children who did not have SCD. They perceived that their children were unhappy This was evident when a caretaker whose child suffered a stroke and cannot walk said;

*“It (worry)comes again especially when I see other people’s children happy and playing…I wonder why I am unfortunate” IDI 6*

All caretakers (12) felt sad when they compared their children’s life span with that of a child without SCD. They all felt their children had a short lifespan and could die any time. This thought was expressed in grief related feelings.

*“I cried so much because I felt my child was not going to grow, she was going to die in the shortest time” IDI 5*

*“I lost strength, felt sad! I did not know what to do because I thought that my child is going to die. I knew sickle cell patients do not live long.” IDI 4*

Having disparity led to experiencing chronic sorrow which is presented below.

### Chronic sorrow

The presence or absence of chronic sorrow was determined from the participants’ responses to the Burke/Eakes Chronic Sorrow Interview Guide (Burke/NCRS) which explores the constructs of chronic sorrow. Nine out of the twelve participants experienced chronic sorrow. For example, one of the caretakers said:

*“When you face challenges, the feelings (grief related feelings) come back, such feelings cannot go completely.” IDI 6*

Chronic sorrow is discussed under two categories namely the nature of Chronic Sorrow and feelings experienced which are presented below. These descriptions are from the nine participants who presented with chronic sorrow.

### Nature of chronic sorrow

Chronic sorrow is known to be pervasive, periodic or permanent and potentially progressive in nature and all these manifestations were articulated by caretakers.

The pervasive nature of chronic sorrow was seen when caretakers reported that having a child who is diagnosed with SCD is demanding and life changing. For some of the caretakers, their entire life was disrupted. This was captured by one of the participants who stays alone with her child because the father of the child abandoned them from the time of diagnosis.

*“Caretakers of children with this disease (SCD), we do not have any time to ourselves...I feel sorry for myself, I can longer go for any outing. I have to be at home all the time” IDI 6*

The participants clearly expressed the periodic and chronicity of this phenomenon by highlighting that the emotions were experienced more than once and recurred as they provided care to the children. The recurrence of the emotions demonstrates the periodic nature of chronic sorrow which was a frequently verbalised assertion by all the nine participants who experienced chronic sorrow. An example of the chronicity of chronic sorrow was seen in a caretaker whose child was diagnosed with SCD a year ago.

*“When my child was diagnosed with SCD I felt terrible and up to now (crying)…I am just trying to accept it but it is very hard to…Anything small takes me back to day one when I got to know that he had SCD” IDI 1*

Another participant who lost three children due to SCD verbalised:

*“I was really upset when I got to know that my child had SCD...even when she falls sick I feel very upset” IDI 6*

The intensity of the emotions that the caretakers experienced reduced over time. The caretakers expressed that through experienced the feeling is periodic. For example, one of the caretakers said;

*“I no longer feel bad to get worried, it(emotions)comes and goes” IDI 3*

### Feelings experienced

The emotions most often re-experienced were worry which was said by 7 out of 9 participants, hopelessness (6 out of 9 participants) which was mentioned twelve times during the course of their interview, emotional pain (5 out of 9 participants) cited fifteen times during the course of their interview, feeling overwhelmed (5 out of 9 participants) cited eleven times during the interviews, sadness (5 out of 9), and distress (5 out of 9). The least frequently re-experienced emotions were anger (1 out of 9), heartbreak (2 out of 9) and fear (3 out of 9).

Parents were worried and afraid that their children were going to die because they have SCD. Others were worried because of the pain and responsibility that come with the disease for example, taking medicine for life, and being hospitalised. A caretaker explained what worries him:

*“I got worried because she is going to be sick more frequently and we shall be in and out of hospital. God willing, she will grow to a certain age because no one with SCD survives for long. I worry because she will be in pain until death” IDI 7*

Other caretakers were worried about producing other children who might have SCD and many of them feared getting pregnant again. This is demonstrated by a caretaker who had two children and one with SCD.

*“Currently I do not feel like having another child because I fear that I could bring another problem (SCD related problem)” IDI 9*

Some of the caretakers felt overwhelmed with the whole situation of having a child with SCD. They felt stressed with the responsibilities that come with having a child with SCD. This feeling was worse if the caretaker had separated from their partner. Some of them felt like giving up sometimes. These feelings can be demonstrated by two caretakers who said:

*“My partner left me because the child had SCD, and this time the child was very sick. I felt the severe pain I experienced in the beginning, I felt dizziness, and got severe headache…sometimes I get overwhelmed, I think to myself, why am I suffering? I should let this child to die.” IDI 6*

*“I was terrified, not knowing what to do…the father of this is not bothered about his welfare.” IDI 4*

Most of the caretakers felt emotional pain and heartbreak because of what they knew regarding SCD and with others it was related to the pain the children go through. This was demonstrated by most of them (8 out of 12) crying during the interviews as they narrated their feelings. This clearly seen in a participant whose child was diagnosed a year ago said the following while crying:

*“It was terrible, up to now. It is so painful. I felt broken, the worst part was when I was admitted at the SCD ward and I saw kids in pain” IDI 1*

Some caretakers felt upset and angry especially those who have had more than one child diagnosed with SCD. These feelings would reoccur when the child was very sick and they felt hopeless. This was vividly expressed by a caretaker who had lost three of her children to SCD.

*“I was really upset, distressed and when she is sick I feel very upset” IDI 11*

Other caretakers felt helpless when chronic sorrow is triggered by other factors. This is clearly stated by a participant who said:

*“I am fat, but when he is in pain, I feel am finished! I have nothing to do and I cannot change the results.” IDI 3*

### Trigger factors

Grief-related feelings were triggered by factors that were categorized as health facility, health worker related, disease related and support related triggers. The most commonly quoted triggers were disease related triggers which were cited forty times, followed by support related triggers mentioned eighteen times and lastly health facility or health worker related triggers cited three times throughout the interviews.

### Disease related trigger factors

The most common disease related triggers included; child being sick (8 out of 9), being uncertain of the future (8 out of 9), and chronicity of the disease (6 out of 10). The least common disease related triggers were thinking of anyone who died of SCD (1 out of 9), lifelong medication given to the child (1 out of 9), and seeing other children in pain or having SCD (3 out of 9).

The chronicity of the disease, with recurrent symptoms which result in hospitalization were the most frequent triggers. Almost all participants reported having grief related feelings when the children were sick. This is captured when a caretaker said;

*“When this child is sick, and I see her health deteriorating, those feelings come back. I feel bad and worry more” IDI 10*

Another caretaker expressed herself this way;

*“When I see him sick with not enough blood, I lose hope. I feel finished and start wondering if he is going to stay alive.” IDI 3*

Giving lifelong medications to the children as well as coming to hospital and sometimes watching other children in pain triggered grief related feelings of some caretakers.

*“Each time I am giving him medication. It is painful, every day when I see the kid taking drugs and he has to take them throughout his life”. IDI 1*

Being uncertain of the future included being uncertain of the child’s health, and future health status of other children who they would give birth to. Caretakers would worry whether the child would live or not and others worried about the erratic nature of the disease. Thoughts like these caused them to have grief related feelings. An example is seen when one of the caretakers whose brother has lost a child to SCD, reported thus;

*“Even when she is not sick, she continues becoming thin even if you feed her….it is not that we are used or strong, we just accepted the condition…. But my brother told me that children with SCD, the end result is death, I should not count her among my children. This worries me” IDI 10*

Another caretaker reported;

*“These children are unpredictable. Now he may be fine and after a few hours he becomes very sick. They are sickly all the time and I worry all the time.” IDI 9*

### Support related trigger factors

The second category includes stigma from the community (55.6%), lack of support from family and partners (22.2%). Stigma from community contributed to caretakers not socializing and experiencing loneliness with no one to share their feelings with. Some of the caretakers chose not to disclose their children’s SCD status to the neighbours because of the fear related to stigma. This was expressed by one of the caretakers:

*“Imagine having this kind of problem and you are not able to tell your neighbours. In my village, if they get to know that your child has SCD, other children would not share with your child even a cup” IDI 10*

Some caretakers’ emotions were triggered by family members or a partner being unsupportive when caring for the child. Some of the family members got tired of supporting the caretakers and gave up. Some of the caretakers were abandoned by their partners because they felt they were responsible for SCD. Others did not want to be associated with anyone with SCD due to the stigma from the community. Two participants in particular noted eleven times in their interviews how this lack of support affected them. Financial constraints worsened the situation. This was verbalised by one of the caretakers whose husband abandoned her;

*“I was very worried, not knowing what to do when he left me with a very sick child. I even thought of buying poison so that my child and I could drink it and die. I was tired of suffering, everything was bad...I didn’t have a job any more, the child was bed ridden, this was the darkness moment in my life.” IDI 6*

One of the caretakers felt the emotional trigger when she read any material regarding SCD.

*“Each time I am reading something and I come across anything to do with sickle cells, it takes me back to day one” IDI 1*

### Health facility related triggers

This was the least cited category that triggered grief related feelings among the caretakers (33.3%). The health facility related triggers included negative attitude of health workers and having the hospital visits.

The health workers communicated rudely to caretakers. This was captured by one of the female caretakers who took her child to an emergency department over the weekend when the child was very sick.

*“In other places, things are different, the health workers are rude and they do not attend to you…. they abuse you…one time I pleaded with her to help me and she said ‘Is she the first one to die? How many corpses do you think pass here?’ I vowed never to go back there” IDI 10*

This was also expressed by another caregiver;

*“They had told us to go and get numbers but on coming back, my son had fainted. I carried the boy to the station were numbers are given before going to the ward. I told the health workers that my child was dying but they replied me rudely that ‘what do you expect us to do with him, look for the doctor’. Can you imagine telling me to look for the doctor yet those nurses were available. It really hurt me…… the situation we go through is not easy” IDI 9*

For the other caretakers, their emotions were triggered by coming to the health facility. A caretaker said;

*“Each time I come here (health facility), I never go back home the same. It takes me time to gather myself and to accept each time I leave the hospital.” IDI 1*

### Coping strategies

Based on the theory of Chronic Sorrow, coping strategies are divided into external and internal sub-themes. External methods were further categorised as community, family, and health facility related support. Internal methods were categorised into behavioural and cognitive coping strategies. The strategies are presented below.

### External coping strategies

#### Family support

This was the most common source of support for all the participants. Family support was received from partners (7 out of 9), parents (5 out of 9), siblings (4 out of 9) and grandparents (1 out of 9). They supported the caretakers emotionally, financially, with counselling, provided housing, and actively got involved in the care of the child with SCD. This is demonstrated in the quotes below given by caregivers:

*“My family members encouraged me by saying I can do it. They encouraged me to seek advice from parents who have children with sickle cell disease and get to know how they have made it.” IDI 10*

*“The good thing their father is supportive financially and emotionally. He says we should take care of the child because with or without the disease they die…He buys whatever is needed even herbal medicine,” IDI 11*

*“My sister really helped me a great deal…Actually she is the one who stood by me…she even provided housing.” IDI 6*

*“What encourages me when she is sick is that when I inform the father, he cares. He helps me so fast to take her to the hospital and so I normally get strength from him. I do not have a job but he supports me financially as well.” IDI 5*

#### Community related support

Community support was mainly received from people in the same situation (66.7%), friends (33.3%) and spiritual leaders (11.1%). They provided emotional support, counselling, and advice to the caretakers.

Caretakers felt better when they met with other caretakers of children with SCD and other chronic illnesses because they shared their stories and this helped them remember that they are not alone in this situation. There were some stories being televised on talk shows where people with SCD shared experiences which educated the public about SCD. These stories encouraged caretakers and they would remember during the re-experience of emotional turmoil that there were other people going through the same situation or even worse. This was illustrated by a one of the caretakers;

*“…sometimes we share out stories and you find that someone has lost children due to SCD and others have about 3 children with SCD… You reflect upon issues you find yourself in a better place and you become stronger” IDI 11*

Another caretaker whose child was diagnosed 1 year ago brought her child to the clinic when he was undergoing sickle cell crisis;

*“I was crying and another caretaker came to me and encouraged me. She showed me a number of kids who had SCD and had grown up. I gained some strength. Then I got a lady who showed me her 34-year-old son with SCD who she still brings to the clinic. I had to accept the situation and be strong.” IDI 1*

The spiritual leaders encouraged one of the caretakers and prayed which gave her hope.

*“My pastor (spiritual leader) gives me hope, he keeps telling me that my son is going to live. He prays for him and says that he will be healed like others and even if there are constraints, I am going to win.” IDI 4*

#### Health facility support

Health facility related support was received from health workers. First, the health facility was an avenue to meet with other people in similar situations and share their stories as described above. Second, health workers from the SCD clinic are friendly to the children and the caretakers, they provide extraordinary care and treatment to the children which gives the caretakers a lot of hope. The health workers also counsel and provide health education for the caretakers which helps them adjust to the new situation in their life. This was acknowledged by all the nine participants.

*“When you enter with a child they first create a good environment for the child by playing and getting to know the child personally. This gave me strength. I saw that doctors care for the children and knew some of them by name” IDI 1*

*“I adjust fast when I take him to the hospital. The way the doctor touches him makes you feel like your child is going to live or rise up again.” IDI 3*

*“The health workers counselled me and taught us about the disease, the causes and how we can care for our children and this made me stronger.” IDI 5*

### Internal management methods

#### Cognitive strategies

The most useful and applied coping strategies were acceptance (8 out of 9), and positive thinking (7 out of 9). The least used were avoidance (4 out of 9), and suppression of emotions (3 out of 9).

Some parents chose to accept the situation and think positively, this helped them to psychologically prepare to fully take on the responsibility of caring for the child. They reported that when they accepted the situation, they were able to comply with the advice and recommendation given by the health workers, which benefited the child.

*“I thought to myself, there are women who are operated and their children die, but this was not the case with me. He will take some years in this world therefore I have to follow instructions of the health workers. Not all children with SCD die, and this helped me to be strong.” IDI 3*

Being knowledgeable about SCD through previous experience within their nuclear or extended family facilitated acceptance. In addition, caretakers whose children were always sickly found it easier to accept different situations.

*“I accept everything, because it is not the first time I am experiencing it. I had a child with SCD but she died. By then I did not know what to do but now I know.” IDI 7*

Others searched for more information regarding SCD so that their understanding of the disease would expand which helped them progress into acceptance.

*“I keep reading more about the disease, I google a lot about it.” IDI 1*

Some caretakers had to accept the situation because they felt helpless.

*“I had to accept it because there is nothing much I can do about it.” IDI 10*

Caretakers who accepted their situation reported feeling inner strength and ready to face anything that came their way. A caregiver reported this after accepting the whole situation.

*“…Inside me, I felt strong for whatever had to come. I felt I have that strength.” IDI 3*

Avoidance was another way in which other caretakers coped. They employed both cognitive and emotional avoidance and behavioural avoidance. Some caretakers (33.3%) kept the child’s health status a secret and this was done due to avoided perceived stigma from the community. Other caretakers avoided interaction with other people (2 out of 9) and kept to themselves because they felt people are tired of them.

*“I do not tell people my problem, some ridicule you…others spread the news to everyone that your child has SCD..., those who are helpful also get tired of you so I go through the situation alone. I decided to isolate myself.” IDI 6*

Suppression of emotions was another way some caretakers coped. They decided not to think about the whole situation and deal with what is at hand.

*“I removed it from my thoughts with the reason that if I keep thinking about it, I will fail to do other things and I might develop pressure (high blood pressure).” IDI 5*

#### Behavioural strategies

These strategies included looking for spiritual interventions (7 out of 9), taking up the responsibility of caring for the child (4 out of 9), developing stronger relationship with the child (2 out of 9).

Spiritual coping included; prayers and trusting in God. Most participants looked to God for strength and hope.

*“I motivate myself by praying and telling God that he is the one who gave me the child with SCD, he has reasons as to why he did that.” IDI 4*

Some caretakers decided to get fully involved in the care of the children, offer the best quality care to the child and spend quality time with the child for as long as they are alive. These parents felt responsible for the care, worked on strengthening their bonds with the children such as considering them as their best friends citing short the life span of the children. Knowing that they are giving the best they can to the children helped them cope with different situations.

*“I have many children, but I call this one (one with SCD) my best friend. I decided to give her my best because children with SCD have a shorter survival rate.” IDI 5*

## Discussion

Caregivers of patients with SCD experience chronic sorrow as described by Eakes et al. (1998). In this study, caretakers experienced different aspects of CS such as disparity, periodic recurrence of emotions which were triggered by numerous factors. Similarly, other researchers have recently documented that caregivers of patients with chronic illnesses experience chronic sorrow [11, 15, 17–20]. For example, a study conducted in Iran among 264 mothers whose children had cancer reported that about 97.7% of the participants either had CS or were more likely to experience [19]. The Kendall scale of chronic sorrow was employed in that study. Likewise, Bowes and colleagues (2009) investigated parents of children who were diagnosed with type 1 diabetes and reported that most of the parents experienced chronic sorrow and were still in denial [11]. The experience of chronic sorrow is high among most caretakers of children/ adults with chronic illnesses, this is because they undergo psychological distress during the time of caregiving. Disparity that is created when the caretakers compare children without SCD to their children results in CS. In the theory of CS, Eakes et al. (1998) explain chronic sorrow as a normal reaction to loss that may be a single episode or an ongoing experience. This elucidates why most caregivers experience CS. Therefore, health workers should recognize that caretakers of children with SCD experience chronic sorrow and help them employ effective coping strategies. Screening for chronic sorrow and supporting caretakers who are experiencing it, is necessary. Although chronic sorrow is viewed as a normal reaction to significant loss, it has been documented that it has the potential to progress to severe psychological disorders such as depression when ineffective coping strategies are employed [9, 21].

In this study population, the most common triggers of grief related feelings emanated from disease related conditions, and perceived lack of support from partners and family members. A few caretakers cited negative attitudes and behaviour of health workers activating sorrow. A number of studies have documented disease related factors which trigger emotional feelings that will never end [13, 18, 22]. For example, Brown and colleagues noted that emotional feelings among caregivers occurred every time the children were sick. Caregivers often worried about their sick children who would never be like other children in terms of growth and daily activities [23]. This is not surprising for caregivers who have children with chronic illnesses because the loss is related to the disease. The diagnosis of the chronic illness is the genesis of the grief related feelings and anytime the illness related health care concerns recurs, they are most likely to experience similar emotions. This calls for strategies to maintain good health among the children. For example, providing affordable preventative treatments that would minimise the number of sickle cell crisis among the children. However, effective coping strategies should be encouraged among the caretakers as well because SCD is a chronic illness and there are a number of uncontrollable factors that may lead to sickle cell crisis.

Close family members were a significant source of social support to the caregivers. Indeed, those who did not get family support experienced more emotional drain especially those whose partners left because of the child’s diagnosis. The lack of social support was linked to misconceptions about the cause of sickle cell disease. This is partly supported by the finding that women were being blamed for the disease. and most of the women were abandoned by their partners after discovering that the child was diagnosed with SCD. Therefore, health education should be provided and reinforced at community level so that society members can understand the disease and why it is important to provide support to the families. Caretakers should be screened so that those who lack social support from partners and other family members can be given more support. Some caregivers reported negative attitude from health workers as a problem to psychosocial support. This has been reported in previous research studies for example, poor communication among health workers was reported by Olwit and colleagues in Uganda [13]. Education in effective communication skills should be included in the training of health workers and continuous medical education sessions should provide methods to help health workers to improve their communication skills.

Both external and internal coping strategies were employed by caretakers as they strived to adjust to the new situation in their life and fulfilling their caregiving roles. Most external support was received from close family members, health workers and caretakers of children with SCD.

Internal strategies were both behavioural and cognitive such as acceptance, positive thinking, getting fully involved in the treatment of the child, avoidance and suppression of emotions. The theory of CS explains that some of these coping strategies are effective while others are not. Acceptance and positive thinking/reframing are among the most effective ways of coping with daily stress [24]. Among the strategies cited by the caretakers, avoidance is known to prolong psychological distress which makes it an ineffective method of coping [25]. Similarly, people who apply emotional suppression have reported higher levels of negative emotions than positive emotions which makes suppression an ineffective way of coping with stress [26]. Moreover, ineffective coping methods increase discomfort among caregivers [8]. This calls for screening of caregivers by health workers and reinforcement of effective coping strategies to help improve the emotional balance and quality of life of caregivers. This also calls for health workers to see caregivers as needing their care as much as their actual patient. This finding also calls for strengthening holistic health care delivered to caregivers of patients with chronic illnesses since the coping strategies used are from social, physical, spiritual, and psychological aspects of life.

For some caretakers, reading about SCD was both a trigger and a coping mechanism. The possible explanation for such an occurrence could be that caretakers want to know as much information about SCD as possible but the findings about SCD are the ones that trigger their emotions as is depicted in our findings that the nature of SCD triggers their emotions. Therefore, as caretakers are given information about SCD, information regarding how to deal with different scenarios should be provided as well. This is emphasized from the results where those who knew about the disease and the interventions had less episodes of emotional turmoil since some triggers were minimised. When events happened, they were assured that it would pass hence shortened the duration of the experience of grief related feelings.

## Conclusion

This study found that caretakers of children with SCD experience chronic sorrow. Chronic sorrow was triggered mainly by disease and support related factors. Therefore, we recommend recognition of CS by health workers through screening. When care givers experiencing CS are identified a support system from family and groups of people in similar situations should be put in place to enhance acceptance through sharing and encouraging each other. This could be part of the ongoing care provided at a specialized SCD clinic. Effective coping strategies such as positive thinking and praying should be encouraged among caretakers to help them cope with the psychological distress.

### Limitation of the study

The local language (Luganda) clumps a number of emotions into one word in English however participants were probed to explain their feelings further and the researchers who were fluent in both languages contextualised the meanings of the words through discussions.

Every caretaker was given a chance to participate on the day of recruitment. This was done to eliminate potential bias in selection of participants.

## Abbreviations

IDI: In depth interview
NCRCS: The Nursing Consortium for Research on Chronic Sorrow
SCD: Sickle cell disease
